# Developing Recommendations to Improve Crisis Line Supports for Public Safety Personnel in Canada: Protocol for a Multimethod National Study

**DOI:** 10.2196/75285

**Published:** 2025-09-26

**Authors:** Gisell Castillo, Chantalle Clarkin, Thiyake Rajaratnam, Fatima Ahmad, Susan Anderson, Gil Angela Dela Cruz, Nadia Aleem, R Nicholas Carleton, Matthew Charbonneau, Adam Crewdson, Danielle Dubé, Max Gomez, Jenny Hardy, Chris Hargreaves, Simon Hatcher, Fardous Hosseiny, Brian Mishara, Eva Serhal, Daisy R Singla, Mark Sinyor, Caitlin Tavares, Karen VanderSluis, Yolanda Wolfgram, Juveria Zaheer, Allison Crawford

**Affiliations:** 1 Centre for Addiction and Mental Health Toronto, ON Canada; 2 Bloomberg Faculty of Nursing University of Toronto Toronto, ON Canada; 3 Trillium Health Parnters, Insight Heath Solutions Toronto, ON Canada; 4 Department of Psychiatry University of Toronto Toronto, ON Canada; 5 Department of Psychology University of Regina Regina, SK Canada; 6 Department of Justice Studies New Brunswick Community College Saint John, NB Canada; 7 Faculty of Continuing Studies Simon Fraser University Burnaby, BC Canada; 8 Richmond Fire Rescue Richmond, BC Canada; 9 Ontario Provincial Police Orillia, ON Canada; 10 Toronto Police Service Toronto, ON Canada; 11 Ottawa Hospital Ottawa, ON Canada; 12 Atlas Institute for Veterans and Families Ottawa, ON Canada; 13 The Institute of Mental Health Research Royal Ottawa Mental Health Centre Ottawa, ON Canada; 14 Université du Québec à Montréal Montréal, QC Canada; 15 Sunnybrook Health Science Centre Toronto, ON Canada; 16 Peel Region Paramedic Services Peel Region, ON Canada

**Keywords:** public safety personnel, crisis lines, suicide prevention, participatory, co-design

## Abstract

**Background:**

Public Safety Personnel (PSP) in Canada experience disproportionately high rates of mental distress and suicidal thoughts and behaviors. PSP mental health is a critical public health issue with far-reaching implications for both individual well-being and the effectiveness of emergency response systems. Crisis lines are an evidence-based public health intervention; however, knowledge gaps remain regarding PSP crisis line use, barriers to accessing services, and the appropriateness of crisis line service models for meeting PSP mental health needs.

**Objective:**

This study aims to address these knowledge gaps using a participatory approach to better understand the crisis line needs and preferences of PSP communities. We also aim to apply our learnings and co-design actionable recommendations for crisis line service improvements and to support PSP who may wish to contact a crisis line.

**Methods:**

This Canada-wide study uses multiple methods across three iterative phases. Phase 1 involves community engagement with PSP to better understand their crisis needs and existing supports. Instrumental to our engagement ethic is the formation of a co-researcher group, composed of PSP with lived experience, who will guide the research process. We will review deidentified crisis line interactions to identify patterns in service use and call outcomes to identify possible points of intervention to enhance service efficacy. We will launch a national web-based anonymous survey to understand the crisis line needs, barriers, and preferences of PSP. Phase 2 focuses on deepening our understanding of PSP experiences with crisis lines through in-depth interviews with those who have accessed or thought about accessing crisis lines and those without crisis line experience who wish to share their views. We will conduct focus groups with crisis sector staff to learn about desired training and resources for improving service delivery to PSP. Phase 3 focuses on developing and conducting co-design workshops to generate evidence-based recommendations with PSP, crisis line responders, researchers, and clinicians. Collaborating across sectors will allow us to codevelop feasible strategies for improving crisis line services to better meet the needs of PSP in crisis who may be inclined to access crisis lines for support.

**Results:**

As of December 2024, the crisis line dataset has been identified and study recruitment for the national survey was completed. Data collection for all other research activities is expected to conclude by May 2025. We anticipate that study findings will be available by the end of 2025.

**Conclusions:**

By identifying barriers to crisis line use and codeveloping solutions, this research will inform policy, service design, and training to enhance services. Ensuring PSP can access crisis line supports that are equitable, evidence-based, and integrated within mental health care systems is crucial to fostering a resilient public safety workforce and emergency response capacities at a societal level.

**International Registered Report Identifier (IRRID):**

DERR1-10.2196/75285

## Introduction

### Background

Urgent attention has been called to the mental health and suicide prevention needs of public safety personnel (PSP; eg, law enforcement, paramedics, and search and rescue) [1] as a critical public health priority. PSP routinely face occupational exposures to potentially traumatic events (PTE) [2-4], operational and organizational stressors [5-8], higher rates of mental health symptoms and disorders [9-12], and higher rates of suicidal thoughts and behaviors than are observed in the general population [13-17].

Much work has been done to develop and evaluate interventions to reduce the mental health impacts of public safety work [18] and mitigate potentially traumatic stress injuries [19] for PSP, with some interventions demonstrating potential benefit. For example, evidence-based psychotherapy (eg, narrative therapy and cognitive behavioral therapy) and resilience training have been associated with reductions in mental health symptoms in PSP [20-24]. Crisis-focused interventions such as critical incident stress management and critical incident stress debriefings [25,26] yield mixed evidence supporting their effectiveness in reducing mental health symptoms. Suicide prevention interventions, however, have rarely been studied among PSP [21,27].

We are aware of three studies (conducted in Canada, South Africa, and the United States) that have sought to evaluate suicide prevention programs for PSP [28-30]. These programs combined multiple intervention components (eg, awareness training, peer support, and critical incident stress management) and included crisis line support services. Of the 3 suicide prevention programs for PSP, 1 study reported longitudinal evidence suggesting a suicide prevention program that included a crisis line for Montreal police was associated with reduced suicide rates over time [28,31]. The contributions of specific program components to reduced suicide rates remain unclear. Peer support programs tailored to police have also been described in the literature, including a helpline for police to access in-person peer support services [32], and a New Jersey-based COP2COP hotline with composite narratives illustrating service user experiences [33].

Given the essential role PSP play in maintaining public safety and emergency response, their mental well-being is a workforce and public health concern, with implications for service continuity, response effectiveness, and broader community health outcomes. Further work is needed to identify and develop appropriate crisis and suicide prevention supports for PSP. There is little research exploring the experiences, needs, or preferences of PSP who are experiencing a mental health or suicide-related crisis. For example, a recent review of qualitative research on first responder well-being found that organizational factors contributed to a decline in well-being rather than exposures to specific events [8]. This suggests there is a knowledge gap about how mental health challenges in PSP arise and develop over time. This knowledge gap is amplified when we consider the numerous barriers to care PSP may face when accessing mental health services, including stigma [24,34-36], mental health literacy, confidentiality concerns, and unsupportive work cultures [37]—factors that may also increase the acuity of a crisis. In summary, there is a dearth of research exploring what PSP need in times of crisis and what supports are most appropriate for PSP experiencing suicidal thoughts and behaviors and mental health crises.

### Crisis Lines and the Launch of 9-8-8: Suicide Crisis Helpline

The primary aim of this study is to identify and address gaps in understanding the experiences of PSP in Canada in crisis and to collaboratively improve available crisis line supports. The launch of Canada’s 9-8-8: Suicide Crisis Helpline on November 30, 2023, represents a significant policy advancement aimed at improving crisis line service availability nationwide [38].

Crisis lines are a key component of Canada’s National Suicide Prevention Action Plan [39,40] and a cornerstone of public health suicide prevention efforts, providing an accessible, scalable, and evidence-based intervention to mitigate mental health crises. They are a readily available intervention with growing evidence supporting their effectiveness at reducing short-term mental distress and suicidal urgency [41-43]. While crisis lines offer an alternative to workplace-based mental health resources, there remains a gap in understanding their accessibility and effectiveness for PSP. Given the structural and attitudinal barriers that PSP may face when seeking mental health support, including stigma, confidentiality concerns, and organizational culture, there is an urgent need to assess whether crisis services meet the acute mental health needs of this population and to ensure that they align with broader public health strategies for suicide prevention.

We aim to generate evidence that informs public health and policy-driven improvements to crisis line services for PSP in Canada. Specifically, we aim to explore the following research questions:

1. What are the needs, experiences, and preferences of PSP related to crisis line services?
2. What are the experiences of crisis line responders when providing services to PSP, and what training and resource supports might improve service provision?
3. What fit-for-purpose tools, strategies, and recommendations can be developed to improve services to provide better support for PSP?

Through a collaborative knowledge translation process, we aim to develop tools, strategies, and policy-relevant solutions to optimize crisis line service delivery for PSP, ensuring that services are accessible, effective, acceptable, and integrated within the broader public health and emergency response system.

### Study Design

**Figure 1:**
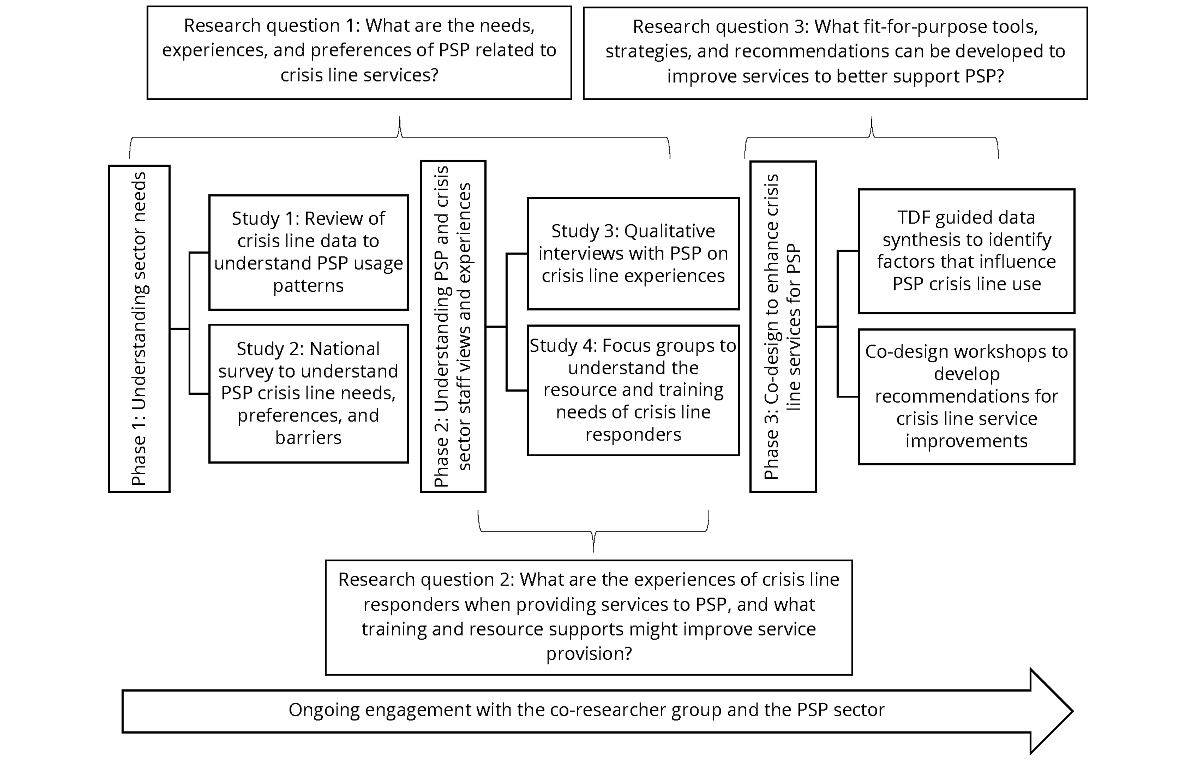
The components of the proposed project. PSP: public safety personnel.

To explore the crisis line needs of PSP, we designed a participatory [44], multimethod study. Figure 1 provides an overview of the study design, including research questions, iterative phases, and methods used.

### Population of Interest

For this study, PSP refers to those who are in professions entrusted with ensuring the safety and security of people in Canada (ie, municipal and provincial police, Royal Canadian Mounted Police, firefighters, paramedics, correctional employees, border services personnel, operational and intelligence personnel, search and rescue personnel, Indigenous emergency managers, and dispatch personnel) [1]. For all research and engagement activities, we include adults (18 years or older) who are current, former, retired, or trainee PSP.

### Participatory Approach

To ensure that our research process is informed by those most affected by our work and leads to meaningful service improvements [45], we are working with a co-researcher group composed of PSP with lived experience with mental illness, suicidal thoughts and behaviors, suicide bereavement, and peer crisis support. Our work is guided by a participatory approach aligned with the public health principles of equity, community engagement, and lived experience leadership, which have been shown to improve the relevance, effectiveness, and implementation of mental health interventions [46,47]. Co-researchers were recruited at the outset of this project by circulating an invitation to collaborate through PSP networks. Specifically, we asked researchers working in the PSP mental health space and PSP mental health organizations to share our invite with their networks. We successfully identified and recruited 5 co-researchers through this approach. An additional co-researcher was recruited through snowball sampling, and the final member was purposively recruited from the crisis sector. In total, 7 co-researchers were onboarded to the team, representing various public safety sectors (law enforcement, fire services, paramedicine, corrections, and the crisis sector) and regions in Canada.

The co-researcher group is an instrumental component of this work, as they provide guidance and feedback on the goals and conduct of the study. Co-researchers collaborate on study design and data collection tools (eg, survey and interview questions), recruitment strategies (eg, text, images, channels), and data interpretation, ensuring that the research reflects the realities of PSP in crisis. Co-researchers are actively involved in interviewing participants, cofacilitating workshops, and developing knowledge translation outputs, including publications and conference presentations. Their contributions strengthen the study’s ability to generate actionable insights that inform policy, crisis service delivery, and public health strategies for suicide prevention. Regular co-researcher meetings will continue throughout the project to promote collaboration, accountability, and lived-experience leadership [48].

Beyond our co-researcher group, we will also engage directly with national, provincial, and municipal PSP organizations (eg, employers, unions, professional associations, and PSP mental health associations) as part of a systems approach to crisis intervention. PSP organizations will be identified through web searches and snowball sampling methods. Outreach meetings and activities will focus on building relationships in the sector, identifying available mental health programs and crisis services for PSP, system-level strengths, gaps, and priorities, refining recruitment strategies, and enhancing knowledge exchange. Outreach activities also support effective knowledge mobilization, allowing findings to be translated into policy and practice improvements at multiple levels of the crisis response system [49].

### Overarching Analytic Framework

This work is guided throughout by the Theoretical Domains Framework (TDF) [50-52]. The TDF synthesizes constructs from 33 theories into 14 domains known to influence behavior: knowledge; skills; social or professional role and identity; beliefs about capabilities; optimism; beliefs about consequences; reinforcement; intentions; goals; memory, attention, and decision processes; environmental context and resources; social influences; emotion; and behavioral regulation. The TDF has been widely used in a health care context to surface barriers and enablers to behavior change [53,54]. The TDF will guide the synthesis of lessons across study phases to facilitate the redesign of crisis interventions by providing a framework with which to identify targets for behavior change. We aim to develop theory-driven strategies that address the identified factors to support behavior change. The strategies will be refined in collaboration with various stakeholders to coproduce recommendations in pursuit of making crisis line services more accessible, appropriate, effective, safe, and equitable for PSP.

## Methods

### Phase 1: Understanding System and Community Needs

#### Study 1: Review of Crisis Line Data to Understand PSP Usage Patterns

To begin exploring questions about the needs, preferences, and experiences of PSP with crisis lines, we first seek to understand PSP crisis line usage patterns, including reasons for contact and interaction outcomes. To this end, we are conducting a retrospective review of existing crisis line data. By reviewing existing crisis line interaction transcripts, we aim to explore and understand the stated reasons that PSP provide for contacting a crisis line, the psychosocial context contributing to their distress in that moment, what they hope to achieve by contacting a crisis line, the narrative of their mental health experience discussed during interactions, the nature of the interventions provided during the interaction (eg, safety planning and resource provision), and the outcomes of interactions (eg, connecting to external resources, follow-up calls, and engaging emergency services).

##### Setting and Background

Talk Suicide Canada was a national suicide prevention crisis line service that provided crisis support to callers and texters. As of November 30, 2023, Talk Suicide Canada was expanded and recreated to become 9-8-8: Suicide Crisis Helpline. Our research team began working in partnership with Talk Suicide Canada to conduct a review of deidentified crisis line call and text interactions that were recorded by Talk Suicide Canada. Per service terms and conditions at the time, it was possible to share deidentified Talk Suicide data with researchers with research ethics approval. The terms of service governing data use came into effect in September 2018. As of November 30, 2023, they were replaced with 9-8-8’s terms of service.

##### Data Characteristics

During crisis line interactions with Talk Suicide Canada, crisis line responders used a client record management system to document text and phone crisis line interaction information. Data that were routinely collected included the date and time of contact, communication modality, length of interaction, phone number, age, gender, province, suicide risk assessment outcomes, reasons for reaching out, responder notes detailing actions taken, and transcripts of interactions. Between May 2022 and April 2023, crisis line responders also had the option of indicating whether interactions were from PSP within the client record management system by selecting “yes,” “no,” or “N/A” to the question “Is the service user a First Responder or Public Safety Personnel?” Based on data retention policies, data for text interactions were available dating back to December 2018, and data for call interactions were available dating back to May 2022.

##### Search Strategy

To identify eligible text interactions between December 2018 and May 2022, Talk Suicide staff were trained to search for applicable text interactions using a set of words related to PSP professions (refer to Multimedia Appendix 1). Applicable call and text interactions occurring between May 2022 and April 2023 were identified based on crisis line responder responses to the question “Is the service user a first responder?” that was embedded in the client record management system. Records identified through these means were reviewed by Talk Suicide staff who were trained to apply the inclusion and exclusion criteria outlined in Textbox 1 to crisis line records.

**Inclusion and exclusion criteria.**

**Inclusion criteria**

- Text interactions were recorded by Talk Suicide Canada in English between December 2018 and April 2023.
- Phone interactions were recorded by Talk Suicide Canada in English between May 2022 and April 2023.
- The texter or caller self-identified as current, former, retired, or a trainee of the public safety personnel (PSP) workforce.

**Exclusion** **criteria**

- The caller or texter was younger than 18 years.
- Interactions were not initiated by PSP.
- Interactions were initiated by PSP on behalf of someone else (third-party calls).

Interaction data that met eligibility criteria were deidentified by Talk Suicide staff by removing personal identifiers and unique details (eg, phone numbers, names, locations, and details that may become identifying through internet searches). Deidentified transcripts and data were then shared with the research team for analysis via secure file transfer.

##### Planned Analyses

Quantitative crisis line data will be analyzed descriptively to generate frequencies, means, and proportions of demographic data (eg, gender, age, and province) and suicidal behaviors and to examine the distribution of suicidal behaviors across PSP groups. Chi-square tests will be run to determine whether there are differences in the rates of suicidal behaviors (ie, current thoughts, past attempts, and current plans) across demographic variables (ie, gender, age, and province), and modality (ie, text or phone) and across PSP groups.

To qualitatively understand the interaction patterns of PSP who contacted the Talk Suicide crisis line, a team of analysts will conduct an inductive content analysis [55] to describe and characterize PSP experiences based on antecedents, behaviors, and experiences during the interaction, and outcomes. Data analysis will be conducted in 6 stages. In stage 1, analysts will read transcripts fully to familiarize themselves with the data. This will be followed by stage 2, an open coding process where descriptive labels will be attached to phrases. Stages 3 and 4 involve reviewing codes for their content (stage 3) and grouping them according to shared characteristics (stage 4). During stage 5, the team of analysts will generate broad categories that describe the interaction process. The research team will meet regularly to discuss different approaches to coding data and to iteratively develop a codebook that outlines the codes and categories generated during stages 3-5. The codebook will both document the analytic process and, once finalized, will represent a shared understanding of the major codes and categories that capture the data [56]. Stage 6 will involve synthesizing and abstracting information from codes and categories to represent the crisis line experience for PSP captured in this sample [57]. The results of this analysis will allow us to describe and characterize how crisis line interactions have unfolded when PSP contacted Talk Suicide Canada. We aim to generate insights on areas for possible service improvements and future research directions based on our findings.

#### Study 2: National Survey to Understand PSP Crisis Line Needs, Preferences, and Barriers

##### Overview

To better understand the needs, preferences, and experiences of a broader sample of PSP, we will conduct a national web-based survey. Crisis lines are an existing suicide prevention and mental health resource that is readily available to all PSP in Canada. However, there may be many reasons why PSP may or may not choose to contact a crisis line during a time of need. We aim to identify what aspects of a crisis line service are most important and meaningful to PSP and to identify barriers and enablers to PSP accessing crisis lines when needed. Using the TDF to identify modifiable factors associated with crisis line use will facilitate the identification of theory-driven strategies [58] to address barriers and enablers to crisis line use. Therefore, we aim to answer the research question: What are the behavioral determinants (TDF domains) of intent to use crisis lines? Based on our understanding of the research literature, we hypothesized (1) social influence, beliefs about consequences, knowledge, and environmental resources and context will be the strongest TDF predictors of whether survey respondents intend to use crisis lines; (2) gender will be associated with intent to use crisis lines, such that men will be less likely to intend to use crisis lines than women or other genders; (3) professions that experience more stigma will be less likely to intend to use crisis lines; and (4) other demographic variables will not be associated with intent to use crisis lines.

##### Survey Development

To identify barriers and enablers to PSP accessing crisis lines, we developed survey questions based on the TDF [59,60]. The TDF domains represent theoretically modifiable factors that influence behavior. Identifying which TDF domains predict behavior, in this case, intent to use crisis lines, can suggest avenues for behavior change interventions. Some TDF domains are more influential than others, depending on the context and behavior in question. Based on outreach meetings with public safety personnel, we know that concerns over engaging emergency services are a major deterrent to accessing crisis line services. Based on existing research, we know that stigma, confidentiality concerns (including concerns that colleagues will find out), not knowing where to get help, and schedule conflicts are common barriers to accessing mental health supports [24,34,35,61].

Therefore, we developed a survey to assess barriers and enablers that correspond to 11 of the 12 original domains (knowledge, skills, beliefs about capabilities, beliefs about consequences, social or professional role and identity, social influences, emotion, memory, attention and decision-making, environmental context and resources, motivation and goals, and intention), as they were the most relevant to this study. The TDF-based questions were designed to reflect the barriers and enablers that have been identified both anecdotally and in the research literature. We also crafted questions about preferences (eg, anonymous, recorded, call answered by peer supporter, and preferred modalities) based on what we learned through outreach meetings with PSP.

To ensure we obtained a comprehensive understanding of the survey sample, we included demographic questions (eg, gender, age, race and ethnicity, and province of residence), work history questions (eg, role, tenure, and status), items exploring past experiences with suicidal thoughts and behaviors, and questions regarding past access to various mental health care services (refer to Multimedia Appendix 2 for the full survey). To ensure items were relevant and understood by PSP and that the length was acceptable, we piloted and tested the survey with co-researchers and research staff before launching.

**Inclusion and Exclusion Criteria**

The national survey was open to all current, former, and trainee members of the Canadian PSP workforce. The survey was available in French and English.

**Recruitment**

The survey was launched in April 2023. To recruit PSP survey participants across Canada, we worked with PSP-serving organizations identified through outreach efforts to disseminate recruitment materials digitally and in person. A variety of tools and strategies were used, including sharing print materials at events and advertising through social media.

**Sample Size**

Our goal was to collect a sufficient number of survey responses to run regression analyses with up to 35 independent variables (ie, TDF domains, demographic variables, and preferences). A minimum required sample size of 277 survey respondents was calculated using the G*power sample size calculation software (version 3.1.9.7; Heinrich-Heine-Universität Düsseldorf), assuming a .05 error probability, .95 power, medium effect size (f^2^=0.15), and 35 degrees of freedom [62]. Based on previous research with PSP samples [9], we expect that approximately 70% of those who begin the survey will complete all sections. Based on this estimated attrition rate, a minimum sample size of 500 survey respondents would yield approximately 350 fully completed surveys, which would allow us to run our planned regression analyses. This estimate aligns with other guidance available for calculating sample size for regression analyses [63,64].

**Analyses**

We will conduct a series of simple linear regression analyses to determine which TDF variables are the strongest predictors of intent to access a crisis line during a time of need. The dependent variable will be based on participants’ responses to a single Likert-scale item: “I would contact a crisis line during a time of need.” Independent variables will include items reflecting the TDF domains (eg, items relating to skills, knowledge, and beliefs about consequences) [59,60]. Simple linear regression will be used to examine the relationship between TDF variables and intent to use crisis lines. Demographic variables will be entered into regression models to control for any potential effects on associations.

This work will allow us to identify possible targets for theoretically derived behaviors and changed strategies.

### Phase 2: Understanding the Views, Experiences, and Practices of PSP and Crisis Sector Staff

#### Study 3: Qualitative Interviews With PSP to Understand Crisis Line Experiences

We seek to conduct in-depth qualitative interviews with PSP who have used or considered using crisis lines to better understand what these experiences mean to PSP and what they see as crucial to change about crisis line services. Semistructured interviews are the preferred method to explore first-person narratives of sensitive topics, as they provide a confidential space for participants to articulate their experience, engage in sense-making, and offer reflections and insights on the phenomena in question [65,66]. Working from a social constructivist paradigm that seeks to understand participant experiences and meaning-making [65], interviews will explore participant thoughts, behaviors, experiences, and sense-making related to mental health crises and the accessibility and adequacy of crisis line services in those pivotal moments.

##### Inclusion and Exclusion Criteria

We are conducting interviews with PSP who have accessed or thought about accessing a crisis line or helpline when experiencing a mental health crisis. We will also seek to conduct semistructured interviews with PSP who may not have any experience accessing crisis lines or helplines but wish to share their views on barriers and enablers to crisis line use and ideas for service improvements.

##### Interview Guide Development

A total of 3 guides have been developed to explore experiences from the following groups: (1) PSP who have contacted a crisis line, (2) those who considered but chose not to contact a crisis line, and (3) those who have not accessed or thought about accessing crisis lines but may have insights to share (refer to interview guide in Multimedia Appendices 3-5). The guides prompt participants to share the chronology, meaning, reflections, and learnings from experiences that can be applied to service improvement considerations. The interview guides have been pilot tested with PSP co-researchers and members of the research team to ensure the questions and interview process are feasible, safe, accessible, and acceptable [67].

##### Recruitment

Interview participants are being recruited by providing a link within the national survey where survey respondents can indicate interest in participating in further research. Additional participants will be recruited by working with PSP-oriented organizations to share posters and advertisements notifying PSP across Canada about this opportunity.

##### Sample Size

Given the specificity of the experiences and views we are sampling, we expect that a small to medium sample size will provide sufficient information power to derive meaningful insights about the data regarding the gaps, barriers, and mental health needs of PSP who access or may want to access crisis lines [68]. Therefore, to achieve sufficient information power, we will conduct 60-80 interviews with PSP across the three groups (accessed a crisis line, thought about accessing a crisis line, and neither accessed nor thought about).

##### Planned Analyses

Interview data will be analyzed using an inductive, reflexive thematic analysis [69-72]. Reflexive thematic analysis allows for the identification of patterns in the data to generate themes that capture participant experiences and sense-making, situating crisis line use in sociocultural contexts, and allows for organizing thematic statements into actionable implications for practice [72]. Analysis will involve generating initial codes, identifying relationships between codes and categories, and generating interpretive themes related to PSP crisis line use, barriers to accessing and using crisis lines, and possible avenues for improving crisis line services. To ensure rigor in the analytic process, a team of analysts will be trained in reflexive thematic analysis and will meet regularly to discuss the coding process and emerging themes. Rather than seeking consensus on coding, the goal of these meetings will be to sense-check, interrogate assumptions, and explore alternative interpretations to enrich the developing analysis [73]. As analyses progress, we will compare emerging themes and findings across the 3 groups to better understand how experiences with crisis lines shape perceptions of mental health resources and the likelihood of contacting a crisis line in the future. Reflections on possible service improvements will also be examined and compared across the 3 groups.

#### Study 4: Focus Groups to Understand the Resource and Training Needs of Crisis Line Responders

##### Overview

Online focus groups are an effective method of data collection when seeking to capture a range of ideas from geographically dispersed participants [74,75]. We will conduct online focus groups with crisis sector personnel connected to the 9-8-8 network to better understand the experiences of crisis line staff when providing services to PSP. We also aim to learn what types of support or resources crisis line responders may need to bolster their capacity for quality service provision to PSP communities.

##### Inclusion and Exclusion Criteria

Participants are required to be 18 years or older and have at least 2 months of experience providing crisis line services. Prior experience providing services to PSP is not required, given that this may be an uncommon experience.

##### Focus Group Guide Development

Focus group participants will be asked about their views and experiences in providing crisis services to PSP, whether additional training and supports tailored to PSP service users would be helpful, and their perspectives on improving services for first responders (refer to the focus group guide in Multimedia Appendix 6).

##### Recruitment

To better understand the views of crisis sector personnel, we aim to recruit a convenience sample of crisis line responders, trainers, and supervisors (volunteer or paid) who work within the 9-8-8 crisis line network. Crisis sector staff will be recruited through an email invitation to be sent out to the 9-8-8 mailing list.

##### Sample Size

We expect crisis line responders working for 9-8-8 to be a more homogenous group, requiring fewer focus groups to generate insights from diverse perspectives. As recommended in the literature, we will aim to conduct between 3 and 5 focus groups with 5-8 participants in each group to ensure sufficient data is collected for the planned analyses [74,76].

##### Planned Analyses

Crisis sector focus groups will be analyzed using an inductive-deductive content analysis [55,57,77,78]. The primary analysis will involve an inductive coding of content categories that describe the perspectives, attitudes, perceived barriers and enablers, sense-making of preliminary data, and ideas for service improvement that each group generates. Data will also be deductively analyzed using the TDF as a guiding framework. The coding and analytic process will be guided by the recommended phases of initialization (initial reading, coding, and abstraction), construction (labeling, classifying, and defining codes and categories), rectification (identifying relationships between codes and categories and with the existing knowledge base, stabilizing themes), and finalization (developing the storyline) to generate meaningful, descriptive themes [78]. We will use a similar approach to the inductive content analysis as described for the crisis line data analysis. The research team will meet to discuss coding and theme generation and will develop a codebook to document the coding and analytic process.

### Phase 3: Co-Design to Enhance Crisis Line Services for PSP

#### Theory-Guided Data Synthesis to Identify Factors That Influence PSP Crisis Line Use

Using the TDF as a guiding framework, results from across all study activities will be analyzed and synthesized according to TDF domains to allow the identification of factors to target for the development of behavior change and implementation strategies. Domains that are consistently represented across crisis line transcripts, survey data, and interview and focus group findings will be prioritized for consideration. Exploration of possible strategies and solutions to TDF-based barriers and enablers will take place during co-design workshops that aim to bridge study findings with actionable recommendations.

#### Study 5: Co-Design Workshops to Develop Recommendations for Crisis Line Service Improvements

Co-design is a dynamic, collaborative, and creative approach to generating solutions to problems identified by communities of interest [47,79]. We will use the generative co-design framework [80] to guide a series of co-design workshops where we will engage project stakeholders in codeveloping recommendations for tools, supportive resources, training modules, and policy changes in support of PSP who may wish to access crisis line services and in support of crisis sector staff who deliver services.

Co-design workshop participants will be engaged in framing the issue so that it reflects a mutual understanding of lived experiences and a vision for the way forward. They will be invited to use generative techniques to surface explicit and latent needs, challenges, and experiences, and to envision alternative future possibilities to address identified issues and challenges. Following the workshops, data from the co-design activities will be analyzed, synthesized, and translated into actionable items. Results from the project will be shared back with participants, and next steps will be articulated and actioned.

##### Participants

We aim to hear a wide range of perspectives during our co-design workshops to ensure that recommendations are grounded in the experiences and needs of those most affected by mental health crises. To achieve this, we will invite PSP with lived experience, mental health care providers, advocates, researchers, and policy makers to ensure that possible recommendations generated reflect the diverse voices and priorities of those impacted by crisis line services and policies. The co-researcher group will play a critical role in the process, contributing to workshop development, facilitating small group discussions, and actively participating as key stakeholders. This inclusive approach is essential for fostering public health solutions that are equitable, relevant, and sustainable.

##### Recruitment

We will advertise co-design workshops to PSP and other contacts who were identified through initial outreach efforts. PSP outreach contacts will receive an email invitation and a request to share the invitation with other PSP in their networks. We will also share newsletters, posters, and advertisements with clinicians and researchers who are known to work with PSP (eg, researchers who are associated with the Canadian Institute for Public Safety Research and Treatment) and with the 9-8-8 network.

##### Sample

We aim to host 3-6 online co-design workshop sessions with 10-30 participants in each session. Hosting multiple sessions will allow us to iteratively build on the insights and recommendations shared in previous sessions. Given that the topic of discussion is highly focused on brainstorming solutions based on study results, we anticipate needing fewer sessions to arrive at a fulsome list of recommendations that reflect the views of various stakeholders [68].

##### Planned Analyses

Similar to focus groups, we will use an inductive-deductive content analysis, guided by the TDF, to summarize findings from co-design workshops and synthesize recommendations.

### Privacy and Confidentiality

Crisis line, interview, focus group, and co-design data will be deidentified. For crisis line data, Talk Suicide staff manually redacted identifying information from transcripts and quantitative data. Deidentified files were shared with the study team using a secure file transfer system that met institutional privacy requirements. The study team assigned unique study numbers to each transcript file. Interview, focus group, and co-design data will be deidentified by transcriptionists and assigned unique study numbers by the study team. Deidentified study data will be kept separate from any identifying source data (ie, recordings and consent forms). The study team will keep a password-protected master log that links study numbers with participant information. Survey data is completely anonymous. Participants were not required to provide any identifying information (eg, name) as part of their participation.

### Compensation Details

For study 1, there was no compensation as there was no direct contact with crisis line callers or texters. For study 2, survey participants were given the option of entering a draw for the chance to win 1 of 5 electronic gift cards valued at CAD $50 (approximately US $36.50). To enter the draw, participants had to provide a valid email address—this information was collected separately from survey data so that personal information remained unlinked to survey data. Once the survey was closed, the study team used a random number generator to identify winners. Participants were then contacted and provided with an e-gift card. For studies 3-5, all participants are provided with a CAD $50 (approximately US $36.50) e-gift card in appreciation of their participation.

### Ethical Considerations

This study has received ethics approval and receives ongoing ethical review from the Centre for Addiction and Mental Health Research Ethics Board (2022-058). All eligible research participants (studies 2-5) will undergo a consent process according to the research ethics board policies. For study 1, deidentified crisis line data was obtained directly from Talk Suicide Canada under the terms of service, which permitted third-party researchers to access and analyze crisis line data if the following conditions were met: (1) the data were deidentified, (2) results were reported in aggregate, (3) research ethics approval was obtained, and (4) the research contributes to improving the quality of crisis line services.

When contacting Talk Suicide Canada, each service user interaction began with a prerecorded greeting, the option to select their language of preference, and a standardized message regarding the potential collection and use of their data for quality improvement before the service user was routed to a responder. The prerecorded voice message, implemented as of August 2019, about data stated, “Your privacy is important to us. Calls may be monitored or recorded for quality control. For more information, visit our website.” Service users who accessed the text service were similarly provided with a link to the Talk Suicide Canada terms of service, which informed users of the potential collection and use of their data for quality improvement and research purposes (implemented as of December 2018).

Given the terms of use, the Centre for Addiction and Mental Health’s legal and privacy offices and the Research Ethics Board determined that additional consent was not necessary for a review of deidentified crisis line data.

## Results

This research project was funded in March 2022. As of December 2024, the crisis line dataset had been identified. In total, 118 crisis line interactions (54 text messages and 64 call interactions) were eligible for inclusion in the study. Data analysis of the crisis line interactions is designed to map out what happens during interactions with PSP so we can identify possible avenues for intervention. The analysis of crisis line transcripts is expected to be completed by July 2025.

Study recruitment for the national survey was launched in April 2023 and concluded on December 31, 2024, with a total sample of 1073. Analysis of survey data has begun and is expected to be completed by July 2025. We facilitated a total of 4 focus groups with crisis sector staff in November 2024. Analysis of the focus group data will commence in March 2025 and will be completed by August 2025. Interview recruitment began in May 2024. As of March 5, 2025, a total of 38 interviews had been conducted. Data collection for all research activities (interviews and co-design workshops) is expected to conclude by June 2025. We anticipate that study findings from all data collected will be available by December 2025 and published in the winter of 2026.

## Discussion

### Summary

To our knowledge, this is the first Canadian study investigating the experiences of PSP with crisis line services on a national scale, addressing a critical gap in the public health literature. This multimethod study will contribute multiple perspectives drawn from community engagement, a review of deidentified crisis line data, a national survey, qualitative interviews, focus groups, and co-design workshops. By leveraging a broad range of data sources, our study will inform both local and national public health strategies to improve access to suicide prevention and crisis line services for PSP and consider whether alternative suicide prevention resources are needed.

Through our collaboration with the co-researchers and other key stakeholders, we will cocreate evidence-based recommendations that reflect the needs of PSP. This participatory, human-centered approach will ensure that our findings resonate with PSP and address their unique challenges. By involving PSP and crisis service providers in every phase of the study, we aim to produce public health interventions that are grounded in equity, accessibility, and effectiveness. The resulting knowledge will guide the development of services that meet the diverse mental health needs of PSP, contributing to system-level improvements in public health and crisis care.

In addition to its national relevance, this study aims to contribute to global suicide prevention efforts by providing an example of how community-engaged research can inform public health interventions. By showcasing a participatory approach that includes PSP as active partners in research, we hope to inspire similar efforts in other countries and settings. This research approach could also be applied across different occupational groups that face exposures to PTE and increased mental health concerns (eg, nurses and physicians) to better understand their crisis line needs and preferences. Our work will provide critical insights into how health care systems can integrate crisis line services for at-risk populations and advance the global public health agenda for suicide prevention and mental health support.

### Limitations

We hope to learn about PSP crisis line needs and preferences through the outlined research. However, we anticipate there will be potential limitations to the data gathered. First, our crisis line data search strategy requires that PSP identify their profession during the crisis interaction. Not all PSP who use the crisis line will reveal their professional backgrounds. Thus, any estimates of call and text frequencies from PSP groups will likely be an underrepresentation. To address this, we have trained responders on the crisis service to more actively take note of when the caller or texter is a PSP. Second, we are focusing PSP contacts on one crisis service, albeit one with many partners and with a national reach, which has an explicit suicide prevention mandate. It may be that other, more general helplines and services, particularly those run by peer supporters, are contacted by PSP more frequently.

Third, our survey and interview data may be susceptible to response bias. Those who take the survey or elect to participate in an interview may overrepresent those with high mental health literacy, awareness of crisis lines, and favorable views toward mental health, help-seeking, and crisis lines. To address this limitation, we will make it explicit during our outreach and engagement efforts that we aim to hear from a diversity of PSP and encourage hearing opposing views and experiences, including those with potentially negative views of mental health and crisis lines. This consideration will also be directly addressed during co-design workshops by acknowledging that the data must be understood with this caveat in mind. Co-design participants will be invited to consider and brainstorm alternative approaches to addressing the crisis and suicide prevention needs of PSP, given that crisis lines may not be used as frequently as other mental health resources. By taking these measures as we conduct our work, we aim to begin building an evidence base from which we can make informed decisions about whether crisis lines are an appropriate resource for PSP communities, whether they need adaptations for these groups, or whether PSP needs are best met through other means. We are confident that even within these constraints, we will gain useful data in an underexplored area.

### Conclusion

PSP play a vital role in maintaining community well-being, yet they face disproportionate mental health risks due to occupational stressors and exposures to potentially traumatic events. Despite the critical need for accessible and effective crisis supports, little is known about how PSP engage with crisis line services, what barriers they face, and how these services can be optimized to meet their needs. This study will generate essential evidence to bridge these gaps, using a participatory, multimethod approach that centers the voices of PSP, crisis line responders, and key stakeholders.

By leveraging community engagement, co-design methodologies, and a robust public health lens, this research will produce actionable, evidence-based recommendations to enhance crisis line services and suicide prevention supports for PSP. Our findings will inform not only service improvements but also broader suicide prevention strategies, ensuring that crisis interventions are accessible, equitable, and responsive to those who need them most. Moreover, by embedding knowledge translation into the research process, we aim to drive real-world change, strengthening crisis response systems, informing policy, and advancing public health efforts to reduce suicide and mental health crises among PSP and supporting the mental well-being of those who serve and protect our communities.

## Appendix

Multimedia Appendix 1 Talk suicide search terms.

Multimedia Appendix 2 National online survey.

Multimedia Appendix 3 Interview guide 1.

Multimedia Appendix 4 Interview guide 2.

Multimedia Appendix 5 Interview guide 3.

Multimedia Appendix 6 Focus group guide.

Multimedia Appendix 7 Peer review report from the Operating Grant Research and Coordination Hubs for Post-Traumatic Stress Injuries in Public Safety Personnel (PTSI in PSP) Committee, Canadian Institutes of Health Research (CIHR, Government of Canada).

## Abbreviations

PSP: public safety personnel
PTE: potentially traumatic event
TDF: Theoretical Domains Framework

## Data Availability

The datasets generated or analyzed during this study are available from the corresponding author on reasonable request.
