# Supplementary material for: Developing Recommendations to Improve Crisis Line Supports for Public Safety Personnel in Canada: Protocol for a Multimethod National Study
Source: JMIR Res Protoc. 2025 Sep 26;14:e75285. doi: 10.2196/75285 (PMC12514416; doi:10.2196/75285)
Supplement: Multimedia Appendix 1 [file resprot_v14i1e75285_app1.docx]

**Appendix A – List of search terms**

1. Emergency responder
2. Firefighter, firefighting, volunteer firefighter
3. Paramedic, EMT, EMS,
4. Parole officer, probation officer, corrections, correctional officer, prison guard, corrections officer
5. Border officer, border services officer, BSO, CBSA
6. Intelligence personnel, intelligence agent, CSIS, secret service
7. Search and rescue, CASARA, SAR, SARVAC
8. Indigenous emergency manager, Métis, First Nations, Inuit, Indigenous
9. Dispatcher, emergency dispatch, 911 operator
10. Police, officer, cop, staff sergeant, detective, sheriff
11. RCMP, Mountie
