# Supplementary material for: Developing Recommendations to Improve Crisis Line Supports for Public Safety Personnel in Canada: Protocol for a Multimethod National Study
Source: JMIR Res Protoc. 2025 Sep 26;14:e75285. doi: 10.2196/75285 (PMC12514416; doi:10.2196/75285)
Supplement: Multimedia Appendix 2 [file resprot_v14i1e75285_app2.docx]

**Appendix B – National Survey**

**Exploring Crisis Services and Suicide Prevention for First Responders and Public Safety Personnel**

**Part 1. Tell us a bit about yourself...**

Please indicate in which public safety sector(s) you have worked. [select all that apply]

RCMP

Municipal police Provincial police Firefighting

Emergency medical services (e.g., paramedic, EMT) Nursing

Corrections Border services

Operations and intelligence Search and rescue

Indigenous emergency management

Public safety communications (e.g., dispatch) Not listed above

Not listed above (please specify):

What is your current employment status?

Active first responder / public safety employee


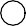

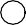

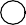

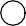

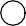

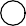


Active first responder / public safety volunteer

First responder / public safety personnel in training

Retired first responder / public safety personnel

On leave from first responder / public safety work

Not listed above

Not listed above (please specify):

How long have you been an active member of the first responder / public safety workforce?

Under 1 year


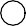

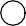

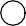

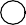

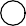


1-10 years

11-20 years

21-30 years

31+

How long were you an active member of the first responder / public safety workforce?

Under 1 year


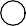

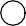

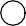

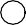

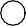


1-10 years

11-20 years

21-30 years

31+

Which best describes your current or most recent first responder / public safety position?

Leadership Administrative Management Frontline Trainee


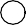

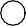

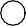

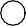

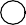

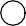


Not listed above

Not listed above (please specify):

What is the average number of hours you work (or worked) as a first responder/public safety personnel per week?

O < 10

O 11-24

O 25-40

O 41-60

O 60+

_________________________________________________________________________________________________

Which best characterizes or characterized your work schedule as a first responder/public safety personnel? [select all that apply]

󠆻 My work schedule is regular from week to week

󠆻 My work schedule is variable from week to week

󠆻 I work day shifts

󠆻 I work evening or night shifts

󠆻 I work a mix of day, evening, and night shifts

󠆻 I work 24-hour shifts

󠆻 I work on call (few scheduled shifts)

󠆻 I work on call in addition to scheduled shifts

󠆻 Not listed above

________________________________________________________________________________________________

Not listed above (please specify):

_____________________________

­­­­­­­­­________________________________________________________________________________________________

What is the highest degree or level of education you have completed?

O Some high school

O High school diploma / General equivalency diploma (GED)

O Some university/college

O Bachelor's degree

O College diploma

O Trade school

O Some post-secondary

O Master's degree

O Academic or professional doctorate degree (Ph.D., MD, or JD) or higher

O Not listed above

O Skip this question

_______________________________________________________________________________________________

Not listed above (please specify):

_____________________________

______________________________________________________________________________________________

Where do you currently live?

Yukon


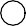

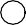

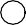

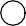

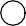

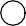

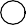

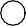

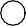

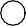

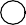

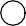

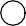


Northwest territories Nunavut

British Columbia Alberta Saskatchewan Manitoba Ontario

Quebec Nova Scotia

New Brunswick Prince Edward Island Newfoundland

How old are you?

18-24


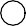

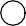

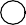

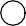


25-45

46-64

65+

Which best describes your gender:

Woman Man


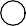

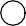

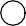

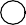

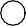

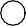

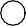

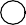

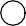


Trans Woman Trans Man

Non-Binary (includes gender fluid, gender queer, androgynous) Two-Spirit

Questioning

Identity not listed (please specify): Skip this question

Identity not listed, please specify:

Please select the category(ies) that best describes your current sexual orientation [select all that apply]:

Asexual Bisexual Demisexual Gay Lesbian Pansexual Queer Questioning

Straight/Heterosexual Two-Spirit

Not listed above Skip this question

Not listed above, please specify:

Which racial or cultural groups best describe you? [Select all that apply]:

Black (African, Afro-Caribbean, African-Canadian descent) East Asian (Chinese, Korean, Japanese, Taiwanese descent) Indigenous (First Nations)

Indigenous (Inuit/Inuk) Indigenous (Métis descent)

Latin American (Hispanic or Latin American descent)

Middle Eastern (Arab, Persian, West Asian descent e.g., Afghan, Egyptian, Iranian, Kurdish, Lebanese, Turkish)

South Asian (South Asian descent e.g., Bangladeshi, Indian, Indo-Caribbean, Pakistani, Sri Lankan) Southeast Asian (Cambodian, Filipino, Indonesian, Thai, Vietnamese, or other Southeast Asian descent) White (European descent)

Identity not listed Skip this question

Identity not listed. Please specify:

Is there anything else you'd like us to know about your identity?

Which of the following best describes where you live?


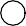


On a reserve

Rural area


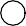

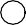

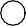

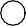

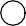


Small population area, with a population of between 1,000 and 29,999 Medium population area, with a population of between 30,000 and 99,999 Large urban population area, with of a population of 100,000 and over.

Skip this question

Which best describes your marital history? [select all that apply]

Married

Living common law

Never married (not living common law) Separated (not living common law) Divorced (not living common law) Widowed (not living common law)

Not listed above Skip this question

Not listed above, please specify:

Which best describes your current marital status?

Married


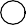

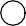

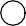

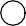

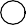

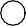

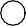

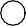


Living common law

Never married (not living common law) Separated (not living common law) Divorced (not living common law) Widowed (not living common law)

Not listed above Skip this question

Not listed above (please specify):

What best describes your household composition? [select all that apply]

Live with spouse (married or common law) Live with family members

Live with extended family members Live with roommates

Live with children Live on my own Not listed above Skip this question

Not listed above (please specify):

Which of the following categories best represents your total household income for the year 2022?

Less than $50,000


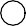

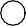

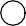


$50,000 and more Skip this question

**Part 2. Experiences with mental health and mental health services**

First responders/public safety personnel experience various occupational and life stressors that are known to impact mental health. Have you experienced any of the following mental health concerns?: [select all that apply]

Acute distress or personal crisis (e.g., feeling intense or overwhelming emotions) that you managed on your own (e.g., with the support of close personal relationships).

Acute distress or personal crisis that required the involvement of an external support or service (e.g., peer support programs, counselling).

Feeling impacted by a potentially traumatic event or work related stressor that you managed on your own. Feeling impacted by a potentially traumatic event or work related stressor that required the involvement of an external support or service.

I have not experienced any of the above mental health concerns. Skip this question

First responders/public safety personnel are more likely to be exposed to potentially traumatic events, experience higher rates of mental health symptoms, suicidal behaviours, and occupational stress injuries than the general public. Suicidal behaviours include thinking about suicide, making a plan to die by suicide and attempting to die by suicide.

Select the option(s) that best describe your experience with suicidal behaviours since becoming a first responder/public safety personnel: [select all that apply]

I have never thought about suicide or experienced any suicidal behaviours I have rarely thought about suicide

I have had thoughts of suicide but no concrete or identifiable plan I have had thoughts of suicide with a concrete plan

I have previously attempted suicide Skip this question

Select the option(s) that best describe your experience with suicidal behaviours in others close to you since becoming a first responder/public safety personnel: [select all that apply]

I have felt concern for a colleague who has mentioned thinking about suicide

I have helped a colleague manage when they were experiencing suicidal behaviours I have experienced the loss of a colleague to suicide

I have felt concern for a friend, family member, or loved one who has mentioned thinking about suicide I have helped a friend, family member, or loved one manage when they were experiencing suicidal behaviours

I have experienced the loss of a friend, family member, or loved one to suicide

I have not encountered a colleague, friend, family member or loved one who was experiencing suicidal behaviours

Skip this question

Now we'd like to learn more about your experiences accessing mental health services.

Have you ever voluntarily accessed mental health services for yourself?

Yes No


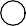

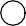


Which of the following services have you voluntarily accessed? [select all that apply]

Mental health professional (e.g., therapist, psychologist, counsellor) Primary care provider (e.g., Family doctor or nurse practitioner) Emergency Department

Group counselling / therapy

Workplace support or Employee Assistance Program (EAP) Peer support helpline (e.g., Boots on the Ground)

Crisis, distress or suicide prevention lines Other helpline

Other mental health service

Mental health professional (e.g., therapist, psychologist, counsellor)

How helpful did you find it?

1 100

*(Place a mark on the scale above)*

Mental health professional (e.g., therapist, psychologist, counsellor) How likely would you be to access this again?

Very Unlikely Unlikely


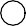

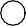

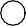

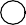

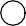


Neither Likely nor unlikely Likely

Very Likely

Primary care provider (e.g., Family doctor or nurse practitioner)

How helpful did you find it?

1 100

*(Place a mark on the scale above)*

Primary care provider (e.g., Family doctor or nurse practitioner) How likely would you be to access this again?

Very Unlikely Unlikely


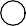

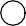

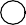

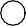

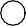


Neither Likely nor unlikely Likely

Very Likely

Emergency Department

How helpful did you find it?

1 100

*(Place a mark on the scale above)*

Emergency Department

How likely would you be to access this again?

Very Unlikely Unlikely


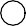

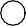

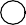

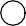

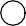


Neither Likely nor unlikely Likely

Very Likely

Group counselling / therapy

How helpful did you find it?

1 100

*(Place a mark on the scale above)*

Group counselling / therapy

How likely would you be to access this again?

Very Unlikely Unlikely


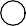

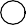

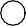

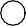

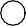


Neither Likely nor unlikely Likely

Very Likely

Workplace support or EAP

How helpful did you find it?

1 100

*(Place a mark on the scale above)*

Workplace support or EAP

How likely would you be to access this again?

Very Unlikely Unlikely


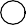

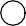

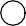

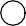

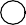


Neither Likely nor unlikely Likely

Very Likely

Peer support helpline (e.g., Boots on the Ground)

How helpful did you find it?

1 100

*(Place a mark on the scale above)*

Peer support helpline (e.g., Boots on the Ground) How likely would you be to access this again?

Very Unlikely Unlikely


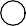

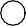

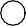

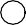

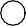


Neither Likely nor unlikely Likely

Very Likely

Crisis, distress or suicide prevention lines

How helpful did you find it?

1 100

*(Place a mark on the scale above)*

Crisis, distress or suicide prevention lines How likely would you be to access this again?

Very Unlikely Unlikely


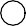

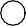

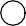


Neither Likely nor unlikely Likely

Very Likely

Other helpline

Please specify type of helpline:

How helpful did you find it?

1 100

*(Place a mark on the scale above)*

How likely would you be to access this again?

Very Unlikely Unlikely

Neither Likely nor unlikely Likely

Very Likely

Other mental health service Please specify type of service:

How helpful did you find it?

1 100

*(Place a mark on the scale above)*

How likely would you be to access this again?

Very Unlikely Unlikely

Neither Likely nor unlikely Likely

Very Likely

**Part 3. Crisis line preferences**

The following questions are about preferences you may have when accessing crisis lines in a time of need. You may answer these questions based on your lived experience or based on what you think best applies to you (hypothetical).

**How important would it be for you...**

To speak with a crisis line responder who is familiar with the nature of first responder / public safety work

To speak with a crisis line responder who understands the technical language of first responder / public safety work

Not at all important

Somewhat unimportant

Neutral Somewhat important

Very important

To speak with a crisis line responder who is familiar with

first responder / public safety sector’s mental health needs

To speak with a crisis line responder who is also a first

Responder/public safety personnel

(peer supporter)

That your call is completely anonymous

That your call or text is not recorded.

To receive a confidential follow up call from a crisis line

responder

To receive a confidential follow

up call from a first responder/public safety personnel (peer supporter)

What would be your preferred way to contact a crisis line:

Phone Online chat Text

I have no preference, all options work for me N/A - I would never contact a crisis line

Is there anything else you'd like us to know about your crisis line needs and preferences?

**Part 4. Barriers and enablers to accessing crisis lines**

Crisis lines are an important mental health and suicide prevention service. Some people may want to access crisis lines but may be hesitant or prevented from doing so for a variety of reasons.

The following questions are about the barriers and enablers you may experience when accessing crisis lines in a time of need. You may answer these questions based on your lived experience or based on what you think best applies to you (hypothetical).

I would be concerned that what I have to say would overwhelm a crisis line responder.

Strongly Disagree

Disagree Neither Agree nor Disagree

Agree Strongly Agree

I think most crisis line interactions end with engaging

emergency services (e.g., calling 911).

I believe my crisis line interaction would be kept

confidential.

I think crisis line responders would understand what I've

been through.

People important to me would think less of me if they knew I

contacted a crisis line.

I would be seen as weak for contacting a crisis line.

My peers would blame me for the problem.

My peers might treat me differently if they knew I

contacted a crisis line.

Contacting a crisis line would harm my credibility as a first

Responder/public safety personnel.

It would be too embarrassing to access a crisis line.

Contacting a crisis line would make me feel worse.

I think I would feel too overwhelmed to contact a crisis

line.

I think I would feel too nervous to contact a crisis line.

I'm not sure what is considered enough of a "crisis" to warrant

contacting a crisis line.

I have heard of the Talk Suicide Canada crisis line (previously Canada Suicide Prevention Service) before.

Strongly Disagree

Disagree Neither Agree nor Disagree

Agree Strongly Agree

I know I can contact Talk Suicide Canada in a time of crisis.

I know where to find information about crisis lines when I need it.

First responders / Public safety personnel

are the type of people to use crisis lines.

I would contact a peer support helpline in a time of need.

I would contact a crisis line during a time of need.

I can recognize when I am experiencing a mental health

crisis.

I have the skills to talk about my mental health with others.

I would be able to use the text and chat functions offered by a

crisis line.

I have a clear plan of how I can access a crisis line when I need

it.

I think contacting a crisis line

would help me manage my mental health needs.

I have a private space where I can access a crisis line.

I have the necessary equipment (e.g., landline, mobile phone,

personal device) to contact a crisis line.

I have access to reliable internet data/connectivity.

I would be able to reach out for help when I need it.

I would be able to contact a crisis line if I needed it.

If I were experiencing a mental health crisis, it would occur to

me to contact a crisis line.

Is there anything else you'd like us to know about barriers (or enablers) to accessing crisis lines?

We are looking for first responders or public safety personnel who would like to share their views on crisis line services in an interview or focus group.

If you think you may be interested in participating in an interview or focus group, please follow this link for more information: <https://redcap.link/aairdfbn>. Please note this is a separate link and your responses to this survey will not be linked to any information you may provide.

Thank you so much for participating in our survey! Below are mental health resources you may find useful.

Mental health resources

If you are in immediate need of help, please contact: 9-8-8 Suicide Crisis Helpline

Call or text 24/7: 9-8-8

Website: https://988.ca/

For other mental health concerns, please contact: Boots on the Ground

Call 24/7: 1-833-677-BOOT (2668)

Website: [https://www.bootsontheground.ca/](http://www.bootsontheground.ca/)

For suicide prevention resources please see: [https://www.suicideinfo.ca/](http://www.suicideinfo.ca/) https://suicideprevention.ca/

For mental health resources for first responders and public safety personnel please see: [https://www.pspmentalhealth.ca/](http://www.pspmentalhealth.ca/) [https://www.pspnet.ca/](http://www.pspnet.ca/)

[https://www.cipsrt-icrtsp.ca/](http://www.cipsrt-icrtsp.ca/)
