# Supplementary material for: Developing Recommendations to Improve Crisis Line Supports for Public Safety Personnel in Canada: Protocol for a Multimethod National Study
Source: JMIR Res Protoc. 2025 Sep 26;14:e75285. doi: 10.2196/75285 (PMC12514416; doi:10.2196/75285)
Supplement: Multimedia Appendix 3 [file resprot_v14i1e75285_app3.docx]

Appendix C - PSP Interview guide 1: Experience with crisis lines or helplines in a time of crisis

Career history

1. I’d love to hear more about your career as a [first responder group]. How did you get started?

Reasons for accessing crisis line services

1. At what point in your career did you *first* connect with a crisis line or helpline?
2. Can you tell me a little bit about why you reached out at that moment in particular? And, please know you do not need to share anything you’re not comfortable talking about or go into details about a painful moment.
3. Can you walk me through how long it took from the moment you realized you needed some mental health support until the moment you reached out to a crisis line or helpline for the first time?
4. In that timeframe, did you talk about your mental health or reach out to anyone in your support network, like family, friends, or trusted colleagues? What was that like for you?
   1. Did you access any other mental health services, resources, or sources of support in that timeframe? What supports did you access?
5. Was there anything that stopped you from reaching out for help during this time? What stopped you?
6. Could you tell me more about the crisis line or helpline service you accessed?
7. Can you walk me through how you decided to contact the crisis line?
8. Thinking back to that moment, was there a reason you chose to access a crisis line as opposed to accessing other resources that may have been available to you? Tell me more about that.

Experiences

1. What was it like for you to contact [crisis line service] in that moment?
2. Was there anything about the interaction that really stuck with you (it could be positive or negative, or just surprising or memorable for another reason)? Tell me more about that.
3. Thinking back to that interaction and the parts that may not have worked so well, were there things that made you feel unsupported, unsafe, or misunderstood? Can you tell me more about that?
4. Was there anything about the interaction that did work well? For example, were there things that made you feel safe, supported, or understood? Can you tell me more about that?
5. Can you tell me a little bit about how the call ended?
6. [If relevant] Can you tell me a bit about the other crisis line services you contacted? How did those experiences compare with what you have shared with me so far?

Outcomes and impacts

1. One of the things we know very little about is what happens after PSP / first responders contact crisis lines. Thinking back to when you contacted [the crisis line service], how did you feel in the moments following the call?
2. How do you feel now about having accessed crisis line services in that moment?
3. Would you access those services again? Tell me more about that.

Opportunities to improve services

1. Was there anything about the interaction you wish had gone differently? [If yes] What do you wish had gone differently?
2. What is one thing you would want a crisis line responder to take away from your experience?
3. What is one thing that would make a difference for you if you were in a crisis and reached out to a crisis line service?
4. Is there anything else you’d like to share about your experience or your thoughts on crisis lines that we haven’t talked about?
