# Supplementary material for: Developing Recommendations to Improve Crisis Line Supports for Public Safety Personnel in Canada: Protocol for a Multimethod National Study
Source: JMIR Res Protoc. 2025 Sep 26;14:e75285. doi: 10.2196/75285 (PMC12514416; doi:10.2196/75285)
Supplement: Multimedia Appendix 4 [file resprot_v14i1e75285_app4.docx]

Appendix D - PSP interview guide 2: Experiences thinking about contacting crisis lines

Career history

1. I’d love to hear more about your career as a [PSP / first responder group]. How did you get started?

Reasons for accessing crisis line services

1. At what point in your career did you first notice you were experiencing challenges with your mental health?
2. Do you remember how long it was from the time you began experiencing challenges with your mental health up until when you first considered contacting a crisis line or helpline?
3. Can you tell me a bit about why you were thinking of reaching out to a crisis line or helpline at that moment in time?
4. During that time, did you talk about your mental health with anyone or reach out to anyone in your support network, like family, friends, or trusted colleagues? What was that like for you?
5. During that time, was there anything that stopped you from reaching out to your support networks or mental health providers for help? Can you tell me more about that?
6. When you were thinking of contacting a crisis line or helpline, did you have a specific service in mind?
   1. [if yes] Could you tell me more about the service you were thinking of accessing?

Experiences

1. We’re interested in understanding what those moments are like for people who do not reach out so that we can consider ways of making crisis lines more accessible to PSP / first responders. Thinking of your experiences where you thought about contacting a crisis line and decided not to, what kinds of concerns came up for you?
   1. Can you walk me through how you decided that contacting a crisis line was not for you?
2. Were there any services, resources, or sources of support that you did seek out in that moment? Can you tell me more about that?
3. Having gone through that experience, how do you feel now about your decision to not contact a crisis line / helpline?
4. Would you consider contacting a crisis line in the future, if you needed to? Tell me about that. Why / why not?

Opportunities to improve services

1. Thinking about your experiences, and first responders more broadly, what is the biggest barrier to first responders accessing a crisis line?
   1. What would you want to see changed about crisis line services to make them more appropriate, accessible, equitable, and safe for all first responders?
2. What would be the most important thing for crisis line responders to understand about first responders so they can provide more effective services?
3. What is one thing that would make a difference for you if you were in a crisis and reached out to a crisis line service?
4. Is there anything else you’d like to share about your experience or your thoughts on crisis lines and first responder mental health that we haven’t talked about?
