# Supplementary material for: Developing Recommendations to Improve Crisis Line Supports for Public Safety Personnel in Canada: Protocol for a Multimethod National Study
Source: JMIR Res Protoc. 2025 Sep 26;14:e75285. doi: 10.2196/75285 (PMC12514416; doi:10.2196/75285)
Supplement: Multimedia Appendix 5 [file resprot_v14i1e75285_app5.docx]

**Appendix E - PSP Interview Guide 3: PSPs without experience with crisis lines**

Section 1: Barriers and enablers to accessing and using crisis lines

1. I’d love to hear more about your career as a [PSP group]. How did you get started?
2. What interested you in this study?
3. From your perspective, where are the gaps in mental health resources for public safety personnel?
4. How are topics like mental health, crises, and suicide talked about in your workplace setting?
5. What are your thoughts on crisis lines? What do you expect to happen when someone reaches out to a crisis line?
6. How appropriate do you think crisis line services, like 9-8-8, are for public safety personnel?
   1. Are there any groups for whom crisis lines might be more or less appropriate?
7. Thinking about yourself and your own experience, how likely would you be to contact a crisis line in a time of need?
   1. What would encourage you to contact a crisis line?
   2. What might stop you from contacting a crisis line?
8. What is one thing that would really make a difference for you if you were in a crisis and reached out to a crisis line service?
9. What are your thoughts on whether your colleagues / people in your PSP profession would contact a crisis line in a time of need?
   1. What might encourage you or [people in your PSP profession] to contact a crisis line?
   2. What might stop your colleagues from contacting a crisis line?
10. What are your thoughts on why some [people in your profession] may choose to contact a crisis line as opposed to other available resources?
11. If you knew someone in [your workplace / colleague / profession] who was experiencing suicidal thoughts or a mental health crisis, how likely would you be to recommend that they access a crisis line?

Section 2: Brainstorming recommendations

1. Thinking about your experiences and public safety personnel more broadly, what is the biggest barrier to public safety personnel accessing a crisis line?
2. What are some other ways we can tackle those barriers? What other ideas do you have for improving crisis line services?
3. What would first responders need to know about crisis line services to feel more confident using them? What is the best way to share this information with first responders?
4. What is important for crisis line staff to understand about first responders / PSP so they can provide more effective services?
5. What would you want to see offered through a national crisis line service, like 9-8-8, so that it is more appropriate for people in your PSP profession?
6. Thank you so much for all your contributions. Is there anything else you would like to share with us today?
