# Supplementary material for: Developing Recommendations to Improve Crisis Line Supports for Public Safety Personnel in Canada: Protocol for a Multimethod National Study
Source: JMIR Res Protoc. 2025 Sep 26;14:e75285. doi: 10.2196/75285 (PMC12514416; doi:10.2196/75285)
Supplement: Multimedia Appendix 6 [file resprot_v14i1e75285_app6.docx]

Appendix F - Crisis sector focus group guide

Experiences

1. When thinking of public safety personnel, we specifically mean callers or texters who fall into one of the following groups: police, RCMP, corrections, border services, dispatch, fire, paramedics, search and rescue, CSIS, and emergency managers for Indigenous communities. Have you ever responded to a call or text from someone who shared that they belong to one of these groups?
2. Has anything about these interactions stood out to you as a whole? Tell me about that. What about these calls or texts has stood out for you?
3. In your experience, are calls from public safety personnel different in any way from calls from other service users?
4. Regardless of how much experience you’ve had speaking with public safety personnel, from your point of view, what challenges would you expect to encounter when providing services to public safety personnel?
5. When you’re working on the lines, does knowing that a caller/texter is public safety personnel change how you approach an interaction or intervention?
6. Based on your own experiences with public safety personnel callers or texters, is there any advice you would have for other crisis line responders regarding how to manage an interaction with public safety personnel?

Training and resource needs

1. We know the role of a crisis line responder is incredibly challenging and requires a very specific skill set. When thinking about crisis line interactions with public safety personnel, what particular tools or skills do you draw on most when supporting these callers/texters?
2. What skills would be helpful to develop to provide better services for public safety personnel?
3. When thinking about crisis line interactions with public safety personnel, what information would be helpful to better understand their experiences?
4. Thinking about the training and resources that you currently have available to you, and recognizing that there are some differences across sites, how well do you feel your current training has prepared you to manage crisis line calls and texts from public safety personnel?
5. What additional training materials and resources do you think would be useful to you for providing effective services to public safety personnel?
6. What would be the best way to deliver training or resources to support crisis line responders with this learning?
7. If cultural competency training were to become available, how interested would you be in taking part?
8. What kind of mental health supports would you want to be put in place to support your well-being?

Improving 988 services

1. Thinking about your own experiences and everything we’ve discussed, what recommendations would you have for 9-8-8 line to better meet the needs of public safety personnel?
2. Thank you so much for sharing your ideas. Is there anything else that would be important for us to know about your thoughts and experiences regarding the crisis line needs of public safety personnel?
