# Supplementary material for: Developing Recommendations to Improve Crisis Line Supports for Public Safety Personnel in Canada: Protocol for a Multimethod National Study
Source: JMIR Res Protoc. 2025 Sep 26;14:e75285. doi: 10.2196/75285 (PMC12514416; doi:10.2196/75285)
Supplement: Multimedia Appendix 7 [file resprot_v14i1e75285_app7.pdf]

Review Type/Type d'évaluation: SO Notes /Notes de l'agent scientifique
Name of Applicant/Nom du chercheur: Crawford, Allison
Application No./Numéro de demande: 475250
Agency/Agence: CIHR/IRSC
Competition/Concours: 2021-10-20 Operating Grant: Research and Coordination Hubs for Post-Traumatic Stress Injuries in Public Safety Personnel (PTSI in PSP)/Subv. de fonctionnement : Centres de recherche et de coordination pour les blessures de stress post-traumatique chez le personnel de la sécurité
Committee/Comité: Op. gr. Res. and Coord. Hubs for Post-Traumatic Stress Injuries in Public Safety Personnel/Sub. de fonc:Centres de recherche et de coordination pour la sécurité publique
Title/Titre: A Theory and Practice Informed Approach to Crisis Intervention for Public Safety Personnel in Canada - Identifying Knowledge Gaps to Inform Action

---

Assessment/Évaluation:

Strengths:

Summary: The committee felt the proposal's goals were important and timely with potential for high impact. Overall they considered the study to be well-designed and to meet a crucial need for support of PSP mental health. Specific strengths include:

- A theory-driven approach guiding data analysis and interpretation
- The expertise and track record of applicants, as well as the institutional and technological structures in place, including support from numerous stakeholder agencies
- Incorporation of lived experience
- Heterogenous population
- Inclusion of 8-10 personnel through entire stream
- Well-developed national coordination /ECHO model
- Mixed design: Combination of qualitative and quantitative methods

Weaknesses:

Summary: Overall the committee thought aspects of the proposal were underdeveloped: In general, if the goal is identifying stressors and barriers to users and support providers there needs to be more reference to existing work on those barriers. A number of methodological details are also lacking, particularly with regard to the quantitative analysis.

Specific concerns about quantitative component:

- No specification of the independent or dependent variables to be used in regression models.
- Details of how the survey will be conducted are lacking. For example: What is the justification for the sample size? How will participants be recruited?
- There is no epidemiologist on the team or specific expertise in the quantitative component
- There are no research questions specified for the quantitative component.
- It is not specified how quantitative findings will be used to inform next steps

There were also some concerns about the qualitative component. For example:

- How will 8-10 people with lived experience be recruited? How will they be stratified to capture

**Review Type/Type d'évaluation:**	SO Notes /Notes de l'agent scientifique
**Name of Applicant/Nom du chercheur:**	Crawford, Allison
**Application No./Numéro de demande:**	475250
**Agency/Agence:**	CIHR/IRSC
**Competition/Concours:**	2021-10-20 Operating Grant: Research and Coordination Hubs for Post-Traumatic Stress Injuries in Public Safety Personnel (PTSI in PSP)/Subv. de fonctionnement : Centres de recherche et de coordination pour les blessures de stress post-traumatique chez le personnel de la sécurité
**Committee/Comité:**	Op. gr. Res. and Coord. Hubs for Post-Traumatic Stress Injuries in Public Safety Personnel/Sub. de fonc:Centres de recherche et de coordination pour la sécurité publique
**Title/Titre:**	A Theory and Practice Informed Approach to Crisis Intervention for Public Safety Personnel in Canada - Identifying Knowledge Gaps to Inform Action

Assessment/Évaluation:

diversity?

- Little space is devoted to specifics of how the qualitative analysis will be conducted

Concerns about buy-in for both interviews and survey

- Asking PSP to self-identify crisis line access may not be feasible due to stigma.
- Self-identification of demographic information (e.g., indigenous identity) may be problematic.
- At this point in time, we are all currently surveyed to saturation. PSP may just not have the time /bandwidth to engage.

Other concerns

- No benchmark criteria for success specified for the crisis line.
- Lack of inclusion/exclusion criteria: There was not a clear definition of who considered under the rubric of PSP for example.
- The snowball sampling method for virtual town halls and interviews can bake in sampling bias.
- There are outstanding questions regard to retrospective reviews of transcripts. For example, how exactly is this information accessed?
- There are outstanding questions about where information on people who have NOT accessed the line is coming from as well as how much information on each individual can be garnered when accessing crisis line.
- Dissemination plans are limited and academic focused. A suggestion was made to recruit and disseminate via magazines/other media commonly accessed by PSP

Budget:

- There was concern about the lack of specific funding for the survey component, given that it is an ambitious undertaking.
